# Corticotropin-releasing hormone, its binding protein and receptors in human cervical tissue at preterm and term labor in comparison to non-pregnant state

**DOI:** 10.1186/1477-7827-4-29

**Published:** 2006-05-31

**Authors:** Aurelija Klimaviciute, Jacopo Calciolari, Emma Bertucci, Susanne Abelin-Tornblöm, Ylva Stjernholm-Vladic, Birgitta Byström, Felice Petraglia, Gunvor Ekman-Ordeberg

**Affiliations:** 1 Dept. of Woman and Child Health, Karolinska Institute, Stockholm, Sweden; 2 Dept. of Pediatrics, Obstetrics and Reproductive Medicine, University of Siena, Siena, Italy

## Abstract

**Background:**

Preterm birth is still the leading cause of neonatal morbidity and mortality. The level of corticotropin-releasing hormone (CRH) is known to be significantly elevated in the maternal plasma at preterm birth. Although, CRH, CRH-binding protein (CRH-BP), CRH-receptor 1 (CRH-R1) and CRH-R2 have been identified both at mRNA and protein level in human placenta, deciduas, fetal membranes, endometrium and myometrium, no corresponding information is yet available on cervix. Thus, the aim of this study was to compare the levels of the mRNA species coding for CRH, CRH-BP, CRH-R1 and CRH-R2 in human cervical tissue and myometrium at preterm and term labor and not in labor as well as in the non-pregnant state, and to localize the corresponding proteins employing immunohistochemical analysis.

**Methods:**

Cervical, isthmic and fundal (from non-pregnant subjects only) biopsies were taken from 67 women. Subjects were divided in 5 groups: preterm labor (14), preterm not in labor (7), term labor (18), term not in labor (21) and non-pregnant (7). Real-time RT-PCR was employed for quantification of mRNA levels and the corresponding proteins were localized by immunohistochemical analysis.

**Results:**

The levels of CRH-BP, CRH-R1 and CRH-R2 mRNA in the pregnant tissues were lower than those in non-pregnant subjects. No significant differences were observed between preterm and term groups. CRH-BP and CRH-R2 mRNA and the corresponding proteins were present at lower levels in the laboring cervix than in the non-laboring cervix, irrespective of gestational age. In most of the samples, with the exception of four myometrial biopsies the level of CRH mRNA was below the limit of detection. All of these proteins could be detected and localized in the cervix and the myometrium by immunohistochemical analysis.

**Conclusion:**

Expression of CRH-BP, CRH-R1 and CRH-R2 in uterine tissues is down-regulated during pregnancy. The most pronounced down-regulation of CRH-BP and CRH-R2 occurred in laboring cervix, irrespective the length of gestation. The detection of substantial expression of the CRH and its receptor proteins, as well as receptor mRNA in the cervix suggests that the cervix may be a target for CRH action. Further studies are required to elucidate the role of CRH in cervical ripening.

## Background

Preterm birth (PTB) is the leading cause of neonatal morbidity and mortality. Despite the existence of strategies for treatment, the frequency of PTB has not changed significantly during the past three decades and the mechanisms underlying the initiation of both preterm and term labor remain largely unknown [1,2]. To date, research and treatment of PTB has focused on myometrial contractions but for successful preterm delivery these contractions must be coordinated with a softening of the cervix. Cervical softening involves intensive remodeling of the extracellular matrix (ECM), with extensive changes in concentration and composition of collagens [3,4] and proteoglycans [5,6]. Cytokines and several other mediators, including estrogen, progesterone, nitric oxide and prostaglandins, participate in the remodeling of extracellular matrix and ripening of the human cervix [1,7-9]. This process can be regarded as an inflammatory reaction associated with elevated levels of cytokines at the time of both preterm and time labor [10-12].

Corticotropin-releasing hormone (CRH) is the principal regulator of the hypothalamic-pituitary-adrenal (HPA) axis. The HPA axis has been of interest in connection with labor since Liggins induced premature parturition in sheep with corticotropin and cortisol infusion [13]. CRH is produced by fetomaternal tissues and secreted into the maternal circulation, so that during pregnancy the maternal plasma levels of this hormone increase while of the corresponding binding protein (CRH-BP) is reduced [14,15]. It has been suggested a "placental clock" model, which determines the duration of gestation and the timing of parturition [16]. Patients at risk for preterm birth have significantly elevated plasma levels of CRH and lower CRH-BP levels [17]. However, usefulness of maternal plasma CRH as a predictor of preterm birth is controversial [18,19].

CRH, CRH-BP and the receptors for CRH, CRH-R1 and CRH-R2, together with the corresponding proteins are expressed in human placenta, deciduas, fetal membranes, endometrium and myometrium [14,20-27]. However, to our knowledge, no studies of a similar nature have been performed on human cervical tissue. Accordingly, our present aims were to compare the levels of expression of CRH, CRH-BP, CRH-R1 and CRH-R2 in human cervical tissue and myometrium in preterm and term labor and not in labor as well as in the non-pregnant state, and to localize the corresponding proteins employing immunohistochemical analysis.

## Methods

### Material

The 67 women included in this study were divided into 5 study groups. The two preterm groups consisted of 14 women undergoing preterm labor (PTL) and 7 at preterm not in labor (PTnotL), delivered by caesarean section prior to the onset of labor. Premature delivery was defined as delivery before the 37^th ^week of gestation. The two term groups included 18 women undergoing term labor (TL) and 21 at term not in labor caesarean section (TnotL). The labor groups were in active labor and demonstrated a ripe cervix, with dilatation more than 4 cm. They were either delivered vaginally or by emergency caesarean section due to malpresentation (at preterm) or due to threatening fetal asphyxia (at term). In all patients delivered by caesarean section, the assessment of cervical dilatation was established immediately before surgery. Women not in labor had unripe cervices (with a Bishop score of <5 points) and were delivered by caesarean section prior to the onset of labor. The preterm indications were suspected ablatio or intra-uterine growth retardation and the term indications were breech presentation, humanitarian or disproportion.

None of the women included in the study suffered from pre-eclampsia, diabetes or other systemic disease, nor did any of our subjects demonstrate clinical signs of infection in connection with parturition or during postpartal period.

There were no significant differences between these four groups with respect to maternal age, parity, previous preterm births and caesarean sections (Table 1).

**Table 1:** Characteristics of the study groups

Parameter	Preterm labor (PTL)	Term labor (TL)	Preterm not in labor (PTnotL)	Term not in labor (TnotL)	Non-pregnant (NP)
Number of subjects	14	18	7	21	7
Age (median, range)	27.5 (18–37)	28.5 (20–37)	32 (27–35)	32 (25–42)	45 (37–49)
Parity (median, range)	1 (0–3)	1 (0–2)	1 (0–2)	1 (0–3)	1 (0–3)
Previous preterm births in the group	1	0	1	2	–
Previous C/S (median, range)	0 (0–1)	0 (0–1)	0 (0–0)	0 (0–2)	–
Gestational age in days (median, range)	237.5 (184–255)	278 (259–292)	193 (183–256)	270 (259–278)	–
Treatment with corticosteroids	4	0	4	0	–

The fifth group consisted of 7 non-pregnant (NP) premenopausal women undergoing hysterectomy for benign gynecological conditions such as menorrhagia or dysmenorrhea (Table 1). One of these patients was receiving replacement therapy with thyroid hormone following thyroidectomy.

Prior to the performance of this study the approval of the local Ethics Committee of Karolinska Institute (Ref. no. 97-089 and 01-395) and the informed consent of each subject was received.

Immediately following parturition, caesarean section or hysterectomy, a biopsy was taken transvaginally (at the 12 o'clock position) from the anterior cervical lip with scissors and tweezers, a technique that our group has applied for 25 years now to obtain cervical tissue samples including squamous and cylindrical epithelium, vessels, glands and extracellular matrix. In the case of caesarian sections, biopsies were also taken from the upper edge of the lower segment incision. Biopsies from non-pregnant myometrium were obtained from the same location and, in addition, from the fundal region. The samples to be utilized for analysis of mRNA were immediately frozen in liquid nitrogen and stored thereafter at -70°C. Biopsies intended for immunohistochemical analysis were rinsed in a physiological saline solution and subsequently fixed in 4% formaldehyde solution for a maximum of 24 hours, followed by dehydration in 70% ethanol and embedment in paraffin.

The limited amount of tissue available from each woman did not allow performance of all of the different analyses on each individual sample.

### Tissue homogenization and extraction of RNA

Tissue homogenization was carried out by the help of a dismembranation apparatus (Retsch KG, Haan, Germany) and total RNA extracted using the Trizol reagent (Invitrogen, Carlsbad, CA, USA) as described previously [12].

### Treatment with DNAse and reverse transcription (RT)

The concentration of total RNA obtained was determined on the basis of the OD_260 _using a DU64^® ^spectrophotometer (Beckman, Palo Alto, CA, USA) and only samples exhibiting an OD_260_/OD_280 _ratio higher than 1.5 were used for RT-PCR. 2 μg total RNA, pre-treated with 2 μl RQ1 RNase-Free DNase (Promega, Madison, WI, USA), was used for RT reaction, which was performed using SuperScript™ RNase H^- ^Reverse Transcriptase (Invitrogen, Carlsbad, California, USA) as described elsewhere [12]. The cDNA was stored at -70°C prior to use.

### Real-Time RT-PCR

To levels of mRNA encoding CRH, CRH-BP, CRH-R1 and CRH-R2 were quantified by real-time RT-PCR employing the Applied Biosystems 7300 Real-Time PCR System (Applied Biosystems, Foster City, CA, USA). All determinations were performed in triplicate in Taqman Universal PCR Master Mix (Applied Biosystems) on 96-well optical PCR plates. Appropriate primers and probes were purchased from commercially available Taqman^® ^gene expression assays (Applied Biosystems). Assay IDs and GenBank Accession numbers: CRH-Hs00174941_m1 (NM_000756), CRH-BP-Hs00181810_m1 (X58022.1), CRH-R1 – Hs00366363_m1 (L23332.1), CRH-R2-Hs01120854_m1 (U34587.1). Ribosomal 18S RNA (X03205.1) was used as an internal standard (Assay ID – Hs99999901_s1, Applied Biosystems).

For each reaction, 5 μl diluted cDNA (corresponding to 20 ng total RNA in all cases except for CRH, where 500 ng total RNA was utilized), 12.5 μl Universal Master Mix, 1.25 μl assay mixture and 18.75 μl sterile water was employed. Real-time PCR reaction was carried out according to a standard manufacturer protocol involving 40–55 cycles of denaturation-annealing. The threshold cycles (C_T_), at which an increase in reporter fluorescence above the baseline signal could first be detected, were determined. The relative levels of the mRNA species of interest were determined employing serial dilutions of the placental (for CRH-BP and CRH-R1) or hippocampal (for CRH-R2) cDNA made from purchased total RNA (Ambion, Austin, TX, USA) and normalizing against the levels of 18S rRNA detected.

### Immunohistochemical analysis

Immunohistochemical analyses were performed on biopsies from 21 subjects: 6 non-pregnant, 10 at term not in labor and 5 at term in labor. In addition to preparation of the biopsies from 16 of these subjects as described above, biopsies from 5 women (1 NP, 2 TnotL, 2 TL) were fixed somewhat differently, i.e., by initial immersion for 2 h at 4°C in a solution containing 4% paraformaldehyde and 14% saturated picric acid in 0.1 M phosphate buffer (pH 6.9), followed by rinsing for at least 24 h in 0.1 M Sörensen's buffer containing 10% sucrose, 0.01% NaN_3 _and 0.02% Bacitracin. Serial cryostat sections 14 μm thick were air-dried.

Tissue sections were stained employing the avidin-biotinylated (ABC)-peroxidase complex method. When analyzed, they were rewashed in PBS for three times five minutes. The endogenous peroxidase activity was eliminated by pre-treatment with 0.3% hydrogen peroxide in methanol for 30 minutes followed by washing in PBS/BSA (0,05%). The slides were incubated overnight with primary rabbit (CRH) or goat (CRH-BP, CRH-R1, CRH-R2) polyclonal antibodies (Santa Cruz Biotechnology Inc, Heidelberg, Germany) directed towards the protein indicated. The dilutions employed for paraffin-embedded sections were as follows: CRH, 1:25 (sc-10718); CRH-BP, 1:100 (sc-1822); CRH-R1, 1:100 (sc-12381); and CRH-R2, 1:50 (sc-1826). In the case of air-dried sections, the antibodies towards CRH were diluted 1:400 and those against CRH-BP 1:800. Subsequently, the sections were washed in PBS/BSA and afterwards incubated with horse anti-goat or horse anti-rabbit the secondary antibody diluted in blocking sera for 30 min. The sections were washed with PBS/BSA, incubated with ABC-complex for 30 minutes, and then rewashed in PBS/BSA. The reaction was developed using the DAB-kit (diaminobenzidine) from Vector (Burlingame, CA, USA). The slides were then rinsed in distilled water. Counterstaining was performed in 10% Mayer's Haematoxylin solution for 3–4 minutes, thereafter the sections were washed in water. Control sections were stained in the same manner, but with omission of the primary antibody. The slides were finally mounted with glycerolgelatin. In all cases immunoreactivity in the squamous and glandular epithelium, the stroma and the vascular endothelium was classified by four independent observers (AK, GEO, BS and JC or EB) employing conventional light-microscopy.

### Statistical analysis

Comparisons between two independent groups were performed utilizing the Mann-Whitney U test. For comparison of cervical and isthmic biopsies from the same patient, the Wilcoxon matched pair test was employed. When more than two groups were compared, the Kruskal-Wallis test was applied, followed by multiple comparison with Bonferroni/Dunn correction. In all cases a p-value of <0.05 was considered to be statistically significant. All calculations were performed with the STATISTICA 6.0 software (StatSoft Inc, Tulsa, OK, USA).

## Results

### mRNA levels in the cervix

#### CRH

Utilizing cDNA corresponding to 20 or 500 ng total RNA for real-time PCR analysis, no CRH mRNA could be detected in any of the cervical samples (data not shown).

#### CRH-BP

**Figure 1:**
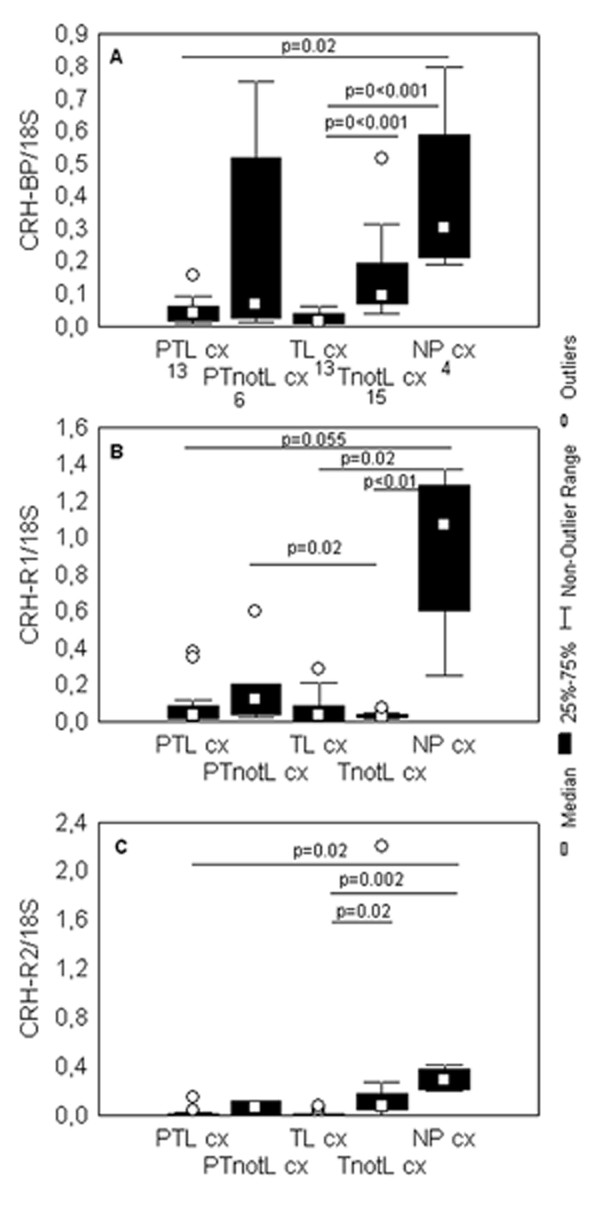
**Levels of CRH-BP, CRH-R1 and CRH-R2 mRNA in the cervical tissue**. Box and whisker plots represent the mRNA levels of (A) CRH-BP, (B) CRH-R1, (C) CRH-R2 in the cervical tissue, normalized to ribosomal 18S mRNA. No CRH mRNA was detected in any of the cervical samples. The studied groups are: preterm labor (PTL), preterm not in labor (PTnotL), term labor (TL), term not in labor (TnotL), non-pregnant (NP). The numbers of samples analyzed for CRH, CRH-BP, CRH-R1 and CRH-R2 in each group are shown under the group name in the part A of the figure. Statistically significant differences are indicated above the plots.

Although, there were no significant differences in the levels of CRH-BP mRNA levels between preterm and term respective groups, we did observe a tendency towards a higher level in the non-pregnant than in the pregnant cervix (Figure 1A). There were significantly higher levels in the non-pregnant cervix compared to PTL (p = 0.02) and TL (p < 0.001) cervix. Furthermore, significantly lower levels of CRH-BP mRNA were seen in the laboring cervix compared to the cervix not in labor at term (p < 0.001). A tendency towards similar difference was in the preterm groups, but this difference was not statistically significant.

Comparing non-pregnant, laboring and non-laboring groups irrespective of gestational age, significantly higher levels of CRH-BP mRNA were detected in the non-pregnant cervix than in the pregnant cervix not in labor (p = 0.03) and in labor (p < 0.001), (Figure 3A). Furthermore, the level of this mRNA was significantly lower in the laboring cervix in comparison to not in labor (p = 0.0001).

**Figure 2:**
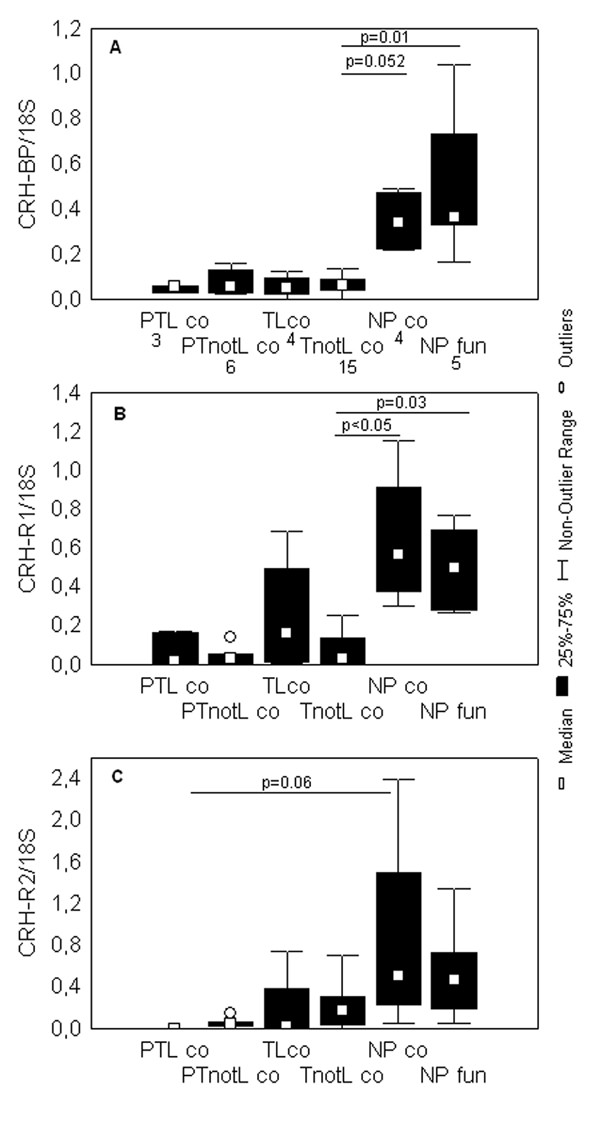
**Levels of CRH-BP, CRH-R1 and CRH-R2 mRNA in the uterine corpus**. Box and whisker plots represent mRNA levels of (A) CRH-BP, (B) CRH-R1, (C) CRH-R2 in the isthmic (co) and fundal (fun) samples, normalized to ribosomal 18S mRNA. The studied groups are: preterm labor (PTL), preterm not in labor (PTnotL), term labor (TL), term not in labor (TnotL), non-pregnant (NP). CRH mRNA was undetectable in most of these samples with the exception of two isthmic samples in PTnoL and two isthmic samples in TnotL groups. The numbers of samples analyzed for CRH, CRH-BP, CRH-R1 and CRH-R2 in each group are shown under the group name in the part A of the figure. Statistically significant differences are indicated above the plots.

**Figure 3:**
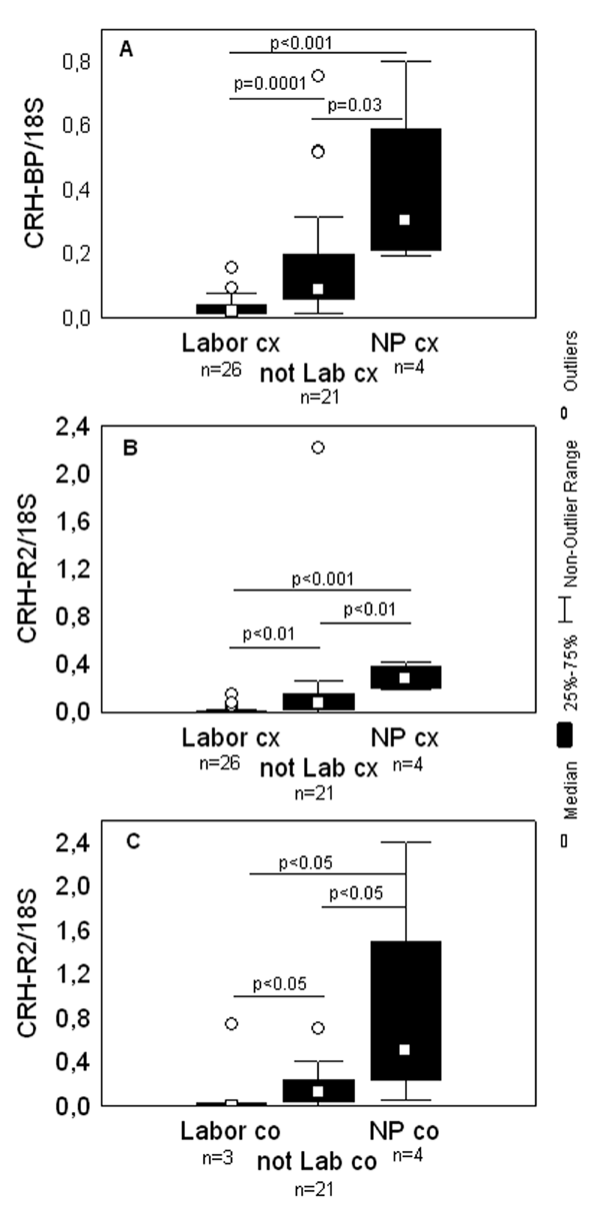
**Levels of CRH-BP and CRH-R2 mRNA in the studied groups irrespective of gestational age**. Box and whisker plots represent mRNA levels of (A) CRH-BP in the cervical tissue (cx), (B) CRH-R2 in the cervical tissue (cx), (C) CRH-R2 in the isthmic biopsies (co) irrespective of gestational age. The studied groups are: in labor (Labor), which includes preterm and term in labor groups; not in labor group (not Lab), which includes preterm and term not in labor groups; non-pregnant group (NP). The number of samples analyzed in each group is shown under the group name. Statistically significant differences are indicated above the plots.

#### CRH-R1

The levels of CRH-R1 mRNA were generally low in all the cervical samples. There were significantly higher levels of CRH-R1 mRNA in PTnotL cervix than in TnotL (p = 0.02) (Figure 1B). As in the case of CRH-BP mRNA, there was a trend towards higher levels of CRH-R1mRNA in non-pregnant tissues compared to pregnant (Figure 1B). The differences were statistically significant between TL and NP cx (p = 0.02), TnotL and NP cx (p < 0.01) and nearly significant between PTL and NP cx (p = 0.055).

Comparing groups irrespective of gestational age, there were no differences in the levels of CRH-R1 mRNA between the laboring and non-laboring groups. The CRH-R1 mRNA expression was significantly higher in the non-pregnant cervix compared to the pregnant cervix not in labor (p < 0.01) and in labor (p < 0.01) (data not shown).

#### CRH-R2

The levels of CRH-R2 mRNA were very low in all the samples. There were no significant differences between preterm and term respective groups. As in the case of both CRH-BP and CRH-R1 mRNA, the CRH-R2 mRNA level was generally higher in the non-pregnant cervix and these differences with respect to the PTL (p = 0.02) and TL (p = 0.002) groups were statistically significant (Figure 1C). Furthermore, there was a significantly higher level of CRH-R2 mRNA in the cervix at term not in labor than in labor (p = 0.02). The same tendency was observed in the preterm groups, but not statistically significant.

When the study groups were compared irrespective of gestational age, significantly lower levels of CRH-R2 mRNA in the laboring (p < 0.01) in comparison to non-laboring cervix were seen (Figure 3B). In line with CRH-BP and CRH-R1 data, the highest expression of CRH-R2 mRNA was in the non-pregnant cervix (Figure 3B).

### mRNA levels in the corpus

#### CRH

When cDNA corresponding to 20 ng total RNA was employed for analysis, no CRH mRNA was detected in any of the samples. When a much larger amount (500 ng) was used, this mRNA species was detected only in two isthmic samples from PTnoL and two isthmic samples from TnotL group (data not shown).

#### CRH-BP

Similar to what was observed in the case of cervix, there were no significant differences between the levels of CRH-BP mRNA comparing preterm and term respective groups (Figure 2A). However, there was a tendency – close to being statistically significant in the case of TnotL (p = 0.052) – towards higher levels of this mRNA species in the non-pregnant isthmus and fundus (which contained the same level) than in the pregnant tissues.

Comparing the CRH-BP mRNA expression between the cervical and isthmic biopsies respectively, significantly higher levels in the TnotL cervix than in the corpus (p = 0.003) were observed. There were no significant differences between the biopsies from corpus in labor and not in labor irrespective of gestational age. In line with the results regarding cervical tissue, there were significantly higher CRH-BP mRNA expression levels in the non-pregnant corpus compared to pregnant not in labor (p = 0.02) and in labor (p = 0.03) (data not shown).

#### CRH-R1

Again as in the case of the cervix, there were no significant differences between the low levels of CRH-R1 mRNA comparing preterm and term groups (Figure 2B). A tendency towards higher levels of CRH-R1 in the non-pregnant corpus compared to pregnant was observed (Figure 2B). The difference was statistically significant between the TnotL and NP corpus (p < 0.05). There were no significant differences between the levels of CRH-R1 comparing samples from cervix, isthmus or fundus.

Comparing groups irrespective of gestational age, there were no differences in the mRNA expression of CRH-R1 between the laboring and non-laboring groups. However, CRH-R1 mRNA levels were significantly higher in the non-pregnant corpus compared to pregnant not in labor (p = 0.0001) (data not shown).

#### CRH-R2

Once again as in the case of the cervix, there were no significant differences between the low levels of CRH-R2 mRNA between preterm and term respective groups. However, there was a tendency – which was nearly statistically significant in the case of PTL (p = 0.06) – towards higher levels of this mRNA species in the non-pregnant isthmus and fundus (which contained the same level) than in the pregnant tissues (Figure 2C). A tendency towards lower levels of CRH-R2 mRNA was seen in the laboring groups compared to the groups not in labor, although this was not statistically significant. There were no significant differences between the samples from the cervix, isthmus or fundus.

Comparing study groups irrespective of gestational age, there were significantly lower levels of CRH-R2 mRNA in the laboring corpus (p < 0.05) compared the corpus not in labor (Figure 3C). As in the case of CRH-BP and CRH-R1 mRNA, the highest level was in the non-pregnant corpus (Figure 3C).

### Immunohistochemical findings

CRH, CRH-BP, CRH-R1 and CRH-R2 proteins were identified in cervix, isthmus and fundus in all study groups. The corresponding negative control sections demonstrated no staining (Figure 6). Similar patterns of staining were obtained with two different fixation procedures. Although immunohistochemistry is not a quantitative method, we want to highlight the following observations.

#### The cervix

**Figure 4:**
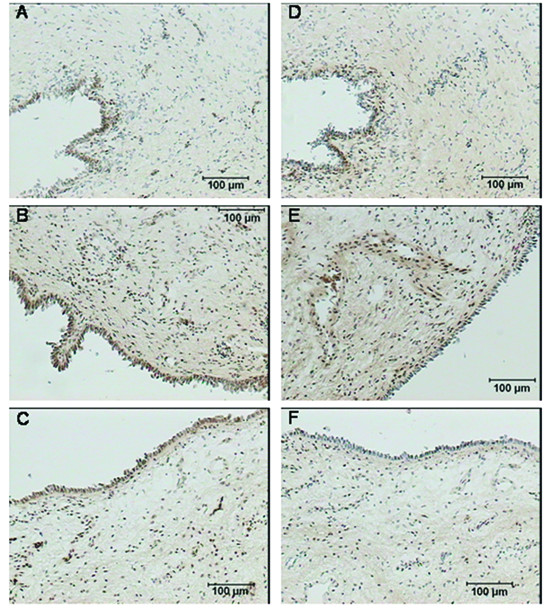
**Immunohistochemical localization of CRH and CRH-BP in the human cervix**. CRH in the cervical epithelium and extracellular matrix in the (A) non-pregnant cervix, (B) term not in labor cervix, (C) term in labor cervix. CRH-BP in the cervical epithelium and extracellular matrix in the (D) non-pregnant cervix, (E) term not in labor cervix, (F) term in labor cervix. Prior to staining, these samples were fixed in a solution of 4% paraformaldehyde and 14% saturated picric acid in 0.1 M phosphate buffer for 2 hours and thereafter rinsed in Sörensen's buffer. The brown color represents positive staining. The magnification is 1 × 100.

**CRH **was detected in the cervical epithelium, fibroblasts and vascular endothelium with weaker staining in the extracellular matrix. This cervical staining was more intense than that in the corpus, with the most pronounced staining at term not in labor (Figure 4A, 4B and 4C).

Staining for **CRH-BP **was weak in the vascular endothelium and the extracellular matrix and even weaker in the cervical epithelium, with no apparent differences between the cervix and the corpus. This staining was weaker in the laboring group compared to the group not in labor (Figure 4D, 4E and 4F).

**Figure 5:**
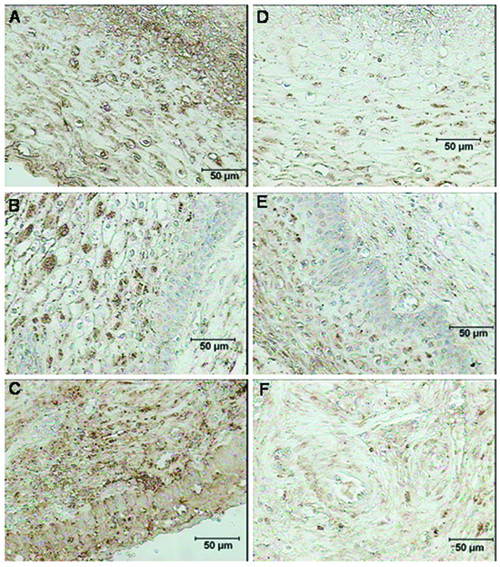
**Immunohistochemical localization of CRH-R1 and CRH-R2 in the human cervix**. CRH-R1 in the cervical epithelium at (A) non-pregnant state, (B) term not in labor, (C) term in labor. CRH-R2 in the (A) cervical epithelium at non-pregnant state, (B) cervical epithelium and extracellular matrix at term not in labor cervix, (C) extracellular matrix at term in labor cervix. These samples were fixed in a 4% formaldehyde solution for a maximum of 24 hours and subsequently dehydrated in 70% ethanol. The brown color represents positive staining. The magnification is 1 × 200.

Staining for **CRH-R1 **was observed in the vascular endothelium and cervical epithelium and heterogeneously in the extracellular matrix (Figure 5A, 5B and 5C). The visual impression was that this staining was more intense in the laboring cervix.

**Figure 6:**
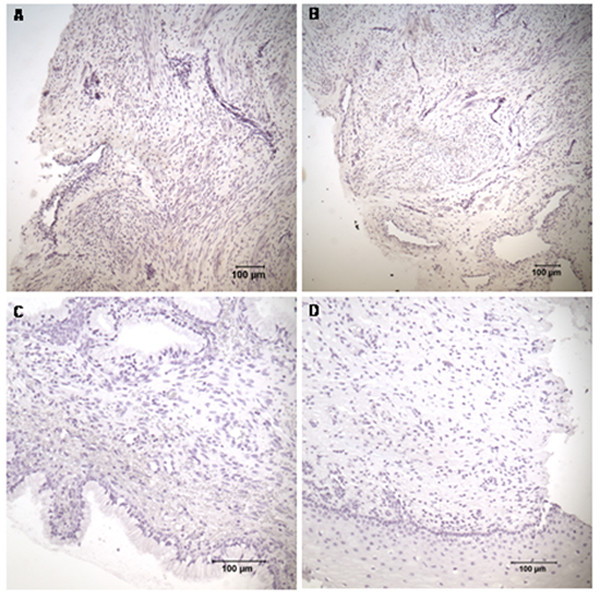
**Negative controls for immunohistochemical staining of the CRH, CRH-BP, CRH-R1 and CRH-R2 in the cervix**. Negative controls for the staining of (A) CRH, (B) CRH-BP, (C) CRH-R1 and (D) CRH-R2. The magnification is 1 × 100 in (A) and (B), 1 × 200 in (C) and (D).

**CRH-R2 **was expressed prominently in the squamous epithelium and vascular endothelium, and more weakly in the extracellular matrix (Figure 5D, 5E and 5F). In this case staining was weaker in labor compared to not in labor group.

#### The corpus (data not shown)

No differences were observed with respect to staining for CRH, CRH-BP, CRH-R1 and CRH-R2 in biopsies from the isthmus and fundus of the same non-pregnant patient, but there were pronounced differences between various individuals within the group.

**CRH **staining was positive in the muscles, fibroblasts and the extracellular matrix with no apparent differences between the different groups.

**CRH-BP **was detected in the muscles, the extracellular matrix and, most prominently, the vascular endothelium, with weaker staining in the laboring group than in the group not in labor, as was also in the case for the cervix.

**CRH-R1 **was expressed predominantly in the vascular endothelium, smooth muscle cells and the extracellular matrix with the most intense staining in the group not in labor.

**CRH-R2 **was observed in the smooth muscle cells, the vascular endothelium and heterogeneously in the extracellular matrix. The staining was weaker in association with labor.

## Discussion

To our knowledge, this is the first report concerning the expression and localization of CRH, CRH-BP, CRH-R1 and CRH-R2 in human cervical tissue.

With respect to CRH, the mRNA levels in the cervix and in most samples from the corpus were below the level of detection, in agreement with earlier studies, in which much lower levels of CRH were found in the myometrium than in the placenta [21]. Of course, we cannot totally exclude the presence of undetectably low levels of CRH mRNA in the cervix and this tissue did stain positively for the CRH protein in connection with our immunohistochemical analysis. However, it appears likely that this cervical CRH originates from the placenta, which is the main source of this protein in reproductive tissues[21].

In present investigation the levels of CRH-BP, CRH-R1 and CRH-R2 mRNA were found to be lower in the pregnant cervix and corpus than in the corresponding tissues from women who were not pregnant. Again, this finding is in line with earlier reports that CRH-R1 is down-regulated in the pregnant in comparison to the non-pregnant myometrium [28,29]. However, there was a discrepancy between these two previous studies: Stevens and coworkers observed up-regulation of CRH-R1 at the time of labor [28], whereas, as was also the case in our study, Rodriguez-Linares et al found no such significant up-regulation [29]. This discrepancy might be due to the use of different methodologies: Stevens et al employed semi-quantitative RT-PCR, Rodriguez-Linares and coworkers competitive RT-PCR and we used real-time RT-PCR, which allows more exact quantitation with high fidelity and identity of the product. The down-regulation of CRH-BP, CRH-R1 and CRH-R2 in the pregnant uterus could be related to the elevated levels of CRH in maternal plasma or, possibly, less CRH effect is needed in pregnancy than in a non-pregnant state. However, our immunohistochemical analysis revealed no clear reduction in the levels of the CRH-BP, CRH-R1 and CRH-R2 proteins in connection with pregnancy, which indicates that there may be no change in physiological function.

There has been much speculation concerning the role of CRH in connection to preterm labor. Here, we observed no differences between preterm and term laboring groups, but the level of CRH-R1 mRNA was significantly higher in preterm not in labor compared to term not in labor cervix. However, eight of our preterm patients received corticosteroid treatment and earlier studies have shown that administration of exogenous corticosteroid enhances the level of maternal plasma CRH, as well as the intensity of immunohistochemical staining for CRH in the placenta and fetal membranes [30,31]. Thus, in order to ascertain whether this treatment influenced our findings, the data was re-analyzed following elimination of the women being treated. The only change was that there were no longer any significant differences between all respective preterm and term groups (data not shown), which indicates that the difference in cervical CRH-R1 mRNA levels between women undergoing preterm and term caesarean section prior to labor was due to corticosteroid treatment. It would have been preferable from a scientific point of view to conduct a similar study on a large number of patients not receiving corticosteroid treatment, but current obstetric practice in Sweden and other Western countries makes it extremely difficult to obtain samples from patients in preterm labor who have not received exogenous corticosteroids.

Since there appeared to be no essential differences between preterm and term groups, we decided to analyze the study groups irrespective of gestational age. This analysis revealed down-regulation of the levels of both CRH-R2 and CRH-BP mRNA in the cervix in connection with labor. This result, in combination with our previous findings that changes in cytokine and 15-hydroxyprostaglandin dehydrogenase levels occur in association with labor irrespective of the length of gestation [12,32], indicate that the process of preterm cervical ripening is similar to the one that occurs at term. This decrease in cervical CRH-BP, both at the mRNA and protein levels at the time of labor is associated with a similar decrease in the maternal plasma [15], which means that more CRH is bioavailable in cervix and can act on the receptors there.

The down-regulation of CRH-R2 in the cervix and myometrium both at mRNA and protein levels at the time of labor is in some respects to the findings by Jirecek and coworkers that expression of the CRH-R2 protein in the myometrium is markedly lower in association with labor at term [33]. However, these other investigators detected no differences in the levels of this protein between patients in preterm labor and not in labor, whereas we report here the same down-regulation tendency at mRNA levels irrespective of gestational age. On the other hand, we performed immunohistochemical analysis only in the term groups and have no data concerning levels of CRH-R2 protein in preterm groups. Similar down-regulation of this receptor has been described in animal studies [34] and this phenomenon may be important for progression of normal labor. Since urocortins are the main ligands for CRH-R2 and play a role in human reproduction [35], it would be important to look for changes in the levels of these hormones in the human cervix during pregnancy and labor.

The detection of substantial expression of the CRH and its receptor proteins, as well as receptor mRNA in the cervix suggests that the cervix may be a target for CRH action. The down-regulation of CRH-R2 and CRH-BP, together with the most intense immunohistochemical staining for CRH-R1 in the cervix at the time of labor indicate that CRH may be involved in cervical ripening. No investigations concerning a possible effect of this hormone on the cervix have been published, but there are several mechanisms by which CRH could be involved in the cervical ripening: by up-regulating expression of NOS [36], enhancing the production of PGE_2 _and PGF_2α _[37] or stimulating secretion of MMP-9 [38]. However, there are controversial findings concerning the role of CRH in the release of cytokines in different tissues [39,40].

Earlier studies from our group have identified fetal fibronectine [41], MMP-8 [42] and Syndican-1 and -3 (unpublished data) in the cervical epithelium. Here, for the first time, we demonstrate the presence of CRH in the cervical epithelium with the highest levels at term not in labor. All these findings support the proposal that the cervical epithelium plays a significant role in the signaling processes involved in cervical ripening.

## Conclusion

CRH-BP, CRH-R1 and CRH-R2 mRNA and the corresponding proteins are expressed in the human cervix. The CRH protein is also detected in the cervical tissue, which indicates that the cervix is a target for the action of this hormone. In comparison to the non-pregnant state, CRH-BP, CRH-R1 and CRH-R2 are all down-regulated in the uterine tissues in connection with pregnancy. The most pronounced down-regulation of CRH-BP and CRH-R2 occurs in the cervix during labor, irrespective of gestational age. Further studies are required to elucidate the role of CRH in the process of cervical ripening.

## Competing interests

The author(s) declare that they have no competing interests.

## Authors' contributions

AK participated in designing this study, performed real-time PCR, statistical analyses, interpretation of data and drafted the manuscript. JC participated in designing the study and performed a part of the immunohistochemical analyses and interpretation of the results. EB also carried out a part of the immunohistochemical analyses and interpretation of the results. SAT selected and recruited patients, collected biopsies and revised the manuscript. YSV selected and recruited patients, collected biopsies and performed critical revision of the manuscript. BB participated in designing the study, the laboratory work and analysis of the data. FP took part in designing of the study and revision of the manuscript. GEO was involved in designing of the study, analysis and discussion of the results and carried out critical revision of the manuscript. All of the authors read and approved the final manuscript.
